# Locational Diversity of Alpha Satellite DNA and Intergeneric Hybridization Aspects in the *Nomascus* and *Hylobates* Genera of Small Apes

**DOI:** 10.1371/journal.pone.0109151

**Published:** 2014-10-07

**Authors:** Sudarath Baicharoen, Takako Miyabe-Nishiwaki, Visit Arsaithamkul, Yuriko Hirai, Kwanruen Duangsa-ard, Boripat Siriaroonrat, Hiroshi Domae, Kornsorn Srikulnath, Akihiko Koga, Hirohisa Hirai

**Affiliations:** 1 Bioscience Program, Faculty of Science, Kasetsart University, Bangkok, Thailand; 2 Center for Advanced Studies in Tropical Natural Resources, National Research University-Kasetsart University, Bangkok, Thailand; 3 Conservation, Research and Education Division, Zoological Park Organization, Bangkok, Thailand; 4 Primate Research Institute, Kyoto University, Inuyama, Aichi, Japan; 5 Chiang Mai Zoo, Chiang Mai, Thailand; 6 Ishikawa Zoo, Tokusan-machi, Noumi, Ishikawa, Japan; 7 Department of Genetics, Faculty of Science, Kasetsart University, Bangkok, Thailand; University of Florence, Italy

## Abstract

Recently, we discovered that alpha satellite DNA has unique and genus-specific localizations on the chromosomes of small apes. This study describes the details of alpha satellite localization in the genera *Nomascus* and *Hylobates* and explores their usefulness in distinguishing parental genome sets in hybrids between these genera. Fluorescence in situ hybridization was used to establish diagnostic criteria of alpha satellite DNA markers in discriminating small ape genomes. In particular we established the genus specificity of alpha satellite distribution in three species of light-cheeked gibbons (*Nomascus leucogenys*, *N. siki*, and *N. gabriellae*) in comparison to that of *Hylobates lar*. Then we determined the localization of alpha satellite DNA in a hybrid individual which resulted from a cross between these two genera. In *Nomascus* the alpha satellite DNA blocks were located at the centromere, telomere, and four interstitial regions. In *Hylobates* detectable amounts of alpha satellite DNA were seen only at centromeric regions. The differences in alpha satellite DNA locations between *Nomascus* and *Hylobates* allowed us to easily distinguish the parental chromosomal sets in the genome of intergeneric hybrid individuals found in Thai and Japanese zoos. Our study illustrates how molecular cytogenetic markers can serve as diagnostic tools to identify the origin of individuals. These molecular tools can aid zoos, captive breeding programs and conservation efforts in managing small apes species. Discovering more information on alpha satellite distribution is also an opportunity to examine phylogenetic and evolutionary questions that are still controversial in small apes.

## Introduction

Introgression and hybridization appear to be frequent mechanisms of speciation in primates [1]. Indeed, field and observation studies have uncovered natural hybrid zones in diverse primate taxa and hybrid offspring are frequent in captivity [2]–[5]. Phylogenic studies have provided evidence for the probable role of hybridization in speciation [6]–[9]. In small apes (gibbons and siamangs), three natural interspecific hybrid zones were documented and intergeneric hybrid offspring were known from captivity. The small apes are distributed throughout Southeast Asia and parts of South and East Asia. Small apes have a higher diversity in morphology, especially pelage patterns, vocalization, and chromosomes than many other primates [3], [10]–[15]. The pelage pattern is often used to identifying species, however, because there is a wide range of pelage characteristics even within a species, classification of taxa on this basis is often quite difficult. It is no exaggeration to state that only specialists in gibbon taxonomy can correctly identify species from pelage alone. We have encountered captive individuals that do not present the exact characteristics of pure species. The genetic traits of such individuals should be checked to identify their species or parental species and it would be helpful if zoological institutions were able to use genetic diagnostic systems to correctly classify species of small apes.

Here we describe chromosome characteristics that function as cytotaxonomic traits that are useful for diagnosing species of small apes. In small apes, chromosomes are expected to be the most significant diagnostic markers for the classification of taxa, because the rate of chromosome change is the highest known in primates. Small apes have a 20 times higher rate of chromosome evolution than the average rate of mammals, excluding rodents (e.g., [16], [17]). Significant diagnostic markers to identify species of small apes currently include chromosome number, translocations, inversions, and the location of constitutive heterochromatin (C-band). Additionally, molecular analyses have also provided significant new insights regarding the phylogeny and distribution of gibbons (e.g., [18], [19]). Comprehensive studies combining molecular phylogenetic and cytogenetic investigations are needed to provide information about the significant evolutionary aspects of gibbons (e.g., [20], [21]).

The small apes have long been classified as a single genus, but then molecular genetics revealed that the four subgenera had similar or greater genetic distances than that between humans and chimpanzees [22]. Therefore, the four subgenera were raised to the taxonomic rank of four separate genera. Presently, small apes are separated into the following four genera, each with a distinct diploid chromosome number: *Hoolock (38)*, *Hylobates (44)*, *Symphalangus (50)*, and *Nomascus (52)* [23]–[27]. As mentioned above, the chromosome evolution of small apes shows a much higher evolutionary rate than most other mammals. The unusual abundance of translocations is one reason for the much higher level of chromosome differentiation [12]. Accordingly, cytogenetic markers are the most useful characteristic to determine the genus and to analyze evolution within this phylogenetic group. In some genera, even species can be distinguished by chromosome differences [11], [20], [28], [29].

Because most lineages of small apes emerged over the short evolutionary time frame of 1.65 million years [30], they can often produce hybrids, even between genera. There have been at least two cases of intergeneric hybrids to date. The first is “Siabon”, a hybrid of the siamang (*Symphalangus*, mother) and gibbon (*Hylobates*, father) [2]. The second is “Larcon” between *Hylobates* (*H. lar*, mother) and *Nomascus* (*N. leucogenys*, father), named from the abbreviation of *lar* gibbons (general term for the genus *Hylobates*) and *con*color gibbons (general term for the genus *Nomascus*) [5]. In both these cases chromosome analysis showed that offspring had chromosome complements originating from two separate genera. This was only possible because the chromosomes coming from the two parents were significantly differentiated and allowed researcher to use these conspicuous chromosomal differences as diagnostic markers. The first case was identified using G- and C-band techniques. The second was identified by chromosome painting, C-banding analyses, and the localization of the nucleolus organizer region. The difference features analyzed reflect the available methods and chromosome technology of the era. A new tool to identify the chromosomes of a genus of small apes is fluorescence in situ hybridization (FISH) with alpha satellite DNA (AS) [31]. Because AS shows specific localization in each genus of small apes [32], it may be a useful tool to identify the parent genera of intergeneric hybrid offspring.

Recently, we found another case of a hybrid offspring between *Nomascus* (mother) and *Hylobates* (father) in Thailand, although the sexes of the parents were opposite from those of the previous case. This case, named “Conlar” because it is a hybrid between the *con*color gibbon and the *lar* gibbon, is the third intergeneric hybrid observed in small apes. We describe here the details of localization of AS in the genera *Nomascus* and *Hylobates* as cytotaxonomic information and characterize the chromosomes of intergeneric hybrid offspring using this information. AS locations in *Nomascus leucogenys* were demonstrated using a FISH technique as described previously [33]. This time, we classified chromosomes harboring AS into three species of the genus *Nomascus,* and determined the parents of the two Larcon and Conlar intergeneric hybrid offspring using this new method combined with previous methods of characterizing chromosomes.

## Methods

### Blood samples and chromosome preparation

Blood samples were collected under anesthetized conditions using ketamine chloride (10 mg/kg) from two hybrid offspring and a female *Nomascus* gibbon at the Chiang Mai Zoo, Thailand, and two (female and male) individuals (ID: 001064376 and 900012000508238) of the genus *Nomascus* and a male individual of *Hylobates lar entelloides* from Thailand (brown) (ID: 90006000007381) at the Khao Kheow Open Zoo, Thailand. Blood samples of the hybrid offspring and non-hybrid individuals were cultured at the Cancer Center, Chulabhorn Hospital, and the Dusit Zoo, Bangkok, Thailand, respectively. Chromosome preparations were made after 70 h of culture, as previously described [34]. Cells were fixed with ethanol and acetic acid (3∶1), and the preparations were transferred to the Primate Research Institute, Kyoto University (KUPRI), Japan, where cytogenetic studies were performed using banding techniques and FISH by SB. Permission to conduct research in Thailand that was approved by the National Research Council of Thailand (NRCT) also included transferring samples collected under the permission. Analyses of FISH with AS obtained from siamangs and chromosome painting with human probes (9, 14, 22, and X from KREATECH Diagnostics, The Netherlands) were performed as described previously [5], [31]. Identifying and numbering chromosomes were performed using 4′,6-diamidino-2-phenylindole (DAPI) banding and counter-staining for FISH analysis because the bands are very similar to the G-bands described previously [28], [35]. The results of FISH and DAPI-bands were imaged using a Zeiss Axioplan 2 mounted systems for cameras (Cool SNAP HQ, Photometrics, USA) and AxioCam MRm (Carl Zeiss, Germany). Image acquisition and processing were performed using IPLab spectrum software (Scanalytics Inc., USA) in Mac OS 9.2 for the former system and Axio Vision 4.8 (Carl Zeiss, Germany) in Windows 7 for the latter system. Ten to twenty chromosome spreads were observed for each sample.

This study was performed in strict accordance with the recommendations in the Guide for the Care and Use of Nonhuman Primates established by the Animal Welfare and Animal Care Committee (Monkey Committee) of the Primate Research Institute, Kyoto University (Primate Research Institute, 2010) and was approved by the Monkey Committee (2011-113). Only blood sampling and photography were performed under anesthesia using ketamine chloride (5–10 mg/kg, intramuscularly). No animals used here were sacrificed. The gibbons used in this study lived in wire netting cages (4 m depth×6 m width×4 m height each) together with a few other individuals. Each cage was additionally equipped with wooden bars, ropes, and a nest box as environmental enrichment. In addition, playing toys were supplied, including a soccer ball, a rubber tire, and ice blocks. Individuals were provided with several types of fruits (mostly bananas, guavas, rose apples, and papayas), vegetables, boiled eggs, and mealworms.

## Results

### Chromosomal localization of alpha satellite DNA in three species of light-cheeked gibbons, the lar gibbon, and their hybrid offspring

**Figure 1:**
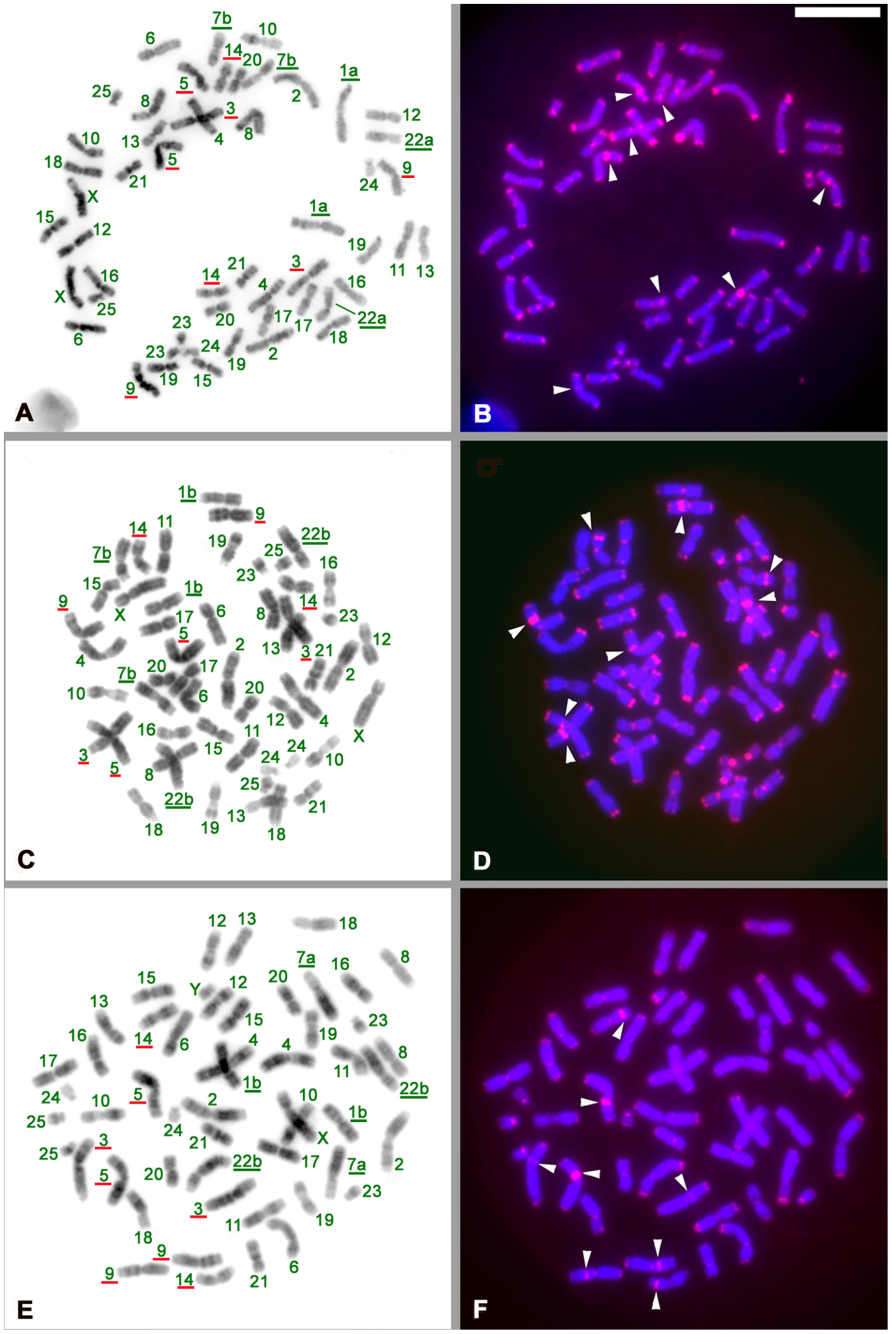
Karyotyping and chromosomal localization of alpha satellite DNA (AS) in three light-cheeked gibbon species. **A** and **B**, *Nomascus leucogenys* (NLE) (female). **C** and **D**, *N. siki* (female) (NSI). E and F, *N. gabriellae* (male) (NGA). **A)**, **C)**, and **E)** DAPI-band (G-like band) reversed from the light fluorescence band of DAPI. **B)**, **D)**, and **F)** Localization of AS (red signal). The number in green is the chromosome number of the genus *Nomascus*. Chromosomes are classified with standard karyotypes as described previously [28]. Numbers and letters with green underlines show the specific karyotype for the species. Numbers with red underlines indicates the chromosome with an interstitial band block in an arm. White arrowheads indicate the location of interstitial AS blocks.

**Figure 2:**
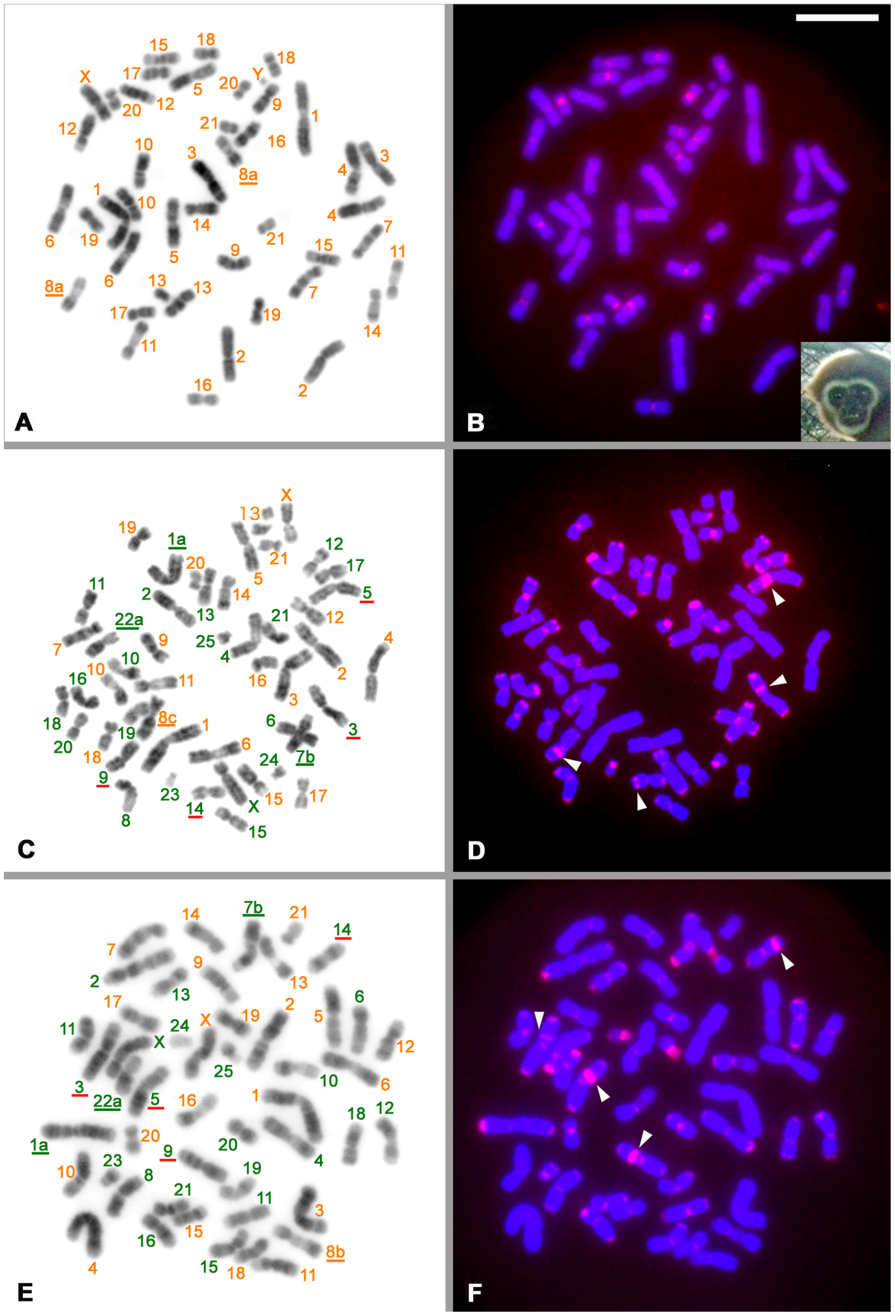
Karyotyping and chromosome localization of AS in *Hylobates lar,* Conlar and Larcon. **A** and **B**, *Hylobates lar entelloides* (male). **C** and **D**, Thai hybrid offspring between *Hylobates lar* (HLA) and *Nomascus leucogenys* (NLE) (female) (NLE/HLA, Conlar). **E** and **F**, Japanese hybrid offspring (female) (HLA/NLE, Larcon). **A), C)**, and **E)** DAPI-band (G-like band) reversed from the light fluorescence band of DAPI. **B), D)**, and **F)** Localization of AS (red signal). Numbers in orange are the chromosome number of the genus *Hylobates*. Chromosomes are classified with standard karyotypes as described previously ([11], [41] for *Hylobates*; [28] for *Nomascus*). Numbers and letters with orange and green underlines show specific karyotype for the species HLA and NLE, respectively. Numbers with red underlines indicate chromosomes with interstitial band blocks in an arm. White arrowheads indicate the location of interstitial AS blocks.

We obtained blood samples from three individuals of the genus *Nomascus* from Thai zoos. Although the three animals were not classified at the species level by morphology, as shown below, chromosome paint analysis revealed that they belonged to the species *Nomascus leucogenys* (NLE), *N. siki* (NSI), and *N. gabriellae* (NGA) by specific inversion morphs. First, to standardize the localization of AS in the three species, we applied FISH analysis using an AS probe on chromosomes of the three species of *Nomascus* (Fig. 1), *Hylobates lar*, and their hybrid offspring (Fig. 2) to observe their characteristics. Chromosomes were identified by DAPI-banding (G-like band) (Fig. 1A, C, E), and then the AS locations were identified on each chromosome (Fig. 1B, D, F). All of the chromosomes had large blocks of AS sequences at the centromeres and telomeres of the three species, NLE, NSI, and NGA. In addition, an interstitial block of AS was observed in the arm of chromosomes 3 (short arm), 5 (long arm), 9 (short arm), and 14 (long arm) of all three species (Fig. 1, chromosome number with red underlines and signal with a white arrow head). These chromosomes and arms were identified by band characteristics as described previously [28].

By contrast, the individual classified as *H. lar entelloides* due to the presence of two homologous chromosome 8a (Fig. 2A) showed a simple pattern. The AS signals, as shown in Fig. 2, were detected only in the centromere regions of 13+X pairs (chromosomes 3, 7∼9, 11, 13∼20 and X) (Fig. 2B). No signals were found in the telomere and interstitial regions, as the case in the genus *Nomascus*. Accordingly, the hybridization signal pattern of AS is a good marker to distinguish between the genera *Nomascus* and *Hylobates* (compare Fig. 1 and 2A).

Based on the hybridization pattern of AS in *Nomascus* and *Hylobates*, we examined the parent species of both cases of Conlar and Larcon hybrids to confirm the presence of both the *Nomascus* and the *Hylobates* genomes in the two hybrid offspring. DAPI bands identified genus-specific karyotypes of NLE 1a-7b-22a and HLA 8c for the Conlar hybrid and NLE 1a-7b-22a and HLA 8b for the Larcon hybrid (Fig. 2C and E). Furthermore, AS-FISH data also identified the *Nomascus* genome in terms of the localizations of centromeres, telomeres, and interstitial regions, and the *Hylobates* genome was confirmed by the simple localization of AS (Fig. 2D and F). The combination of AS locations and DAPI bands made it easy to distinguish the genomes of parent species in intergeneric hybrid offspring (Fig. 2C–F).

### Cytotaxonomic identification of species in the taxa of light-cheeked gibbons and an intergeneric hybrid offspring by FISH analyses with human chromosome paint probes

**Figure 3:**
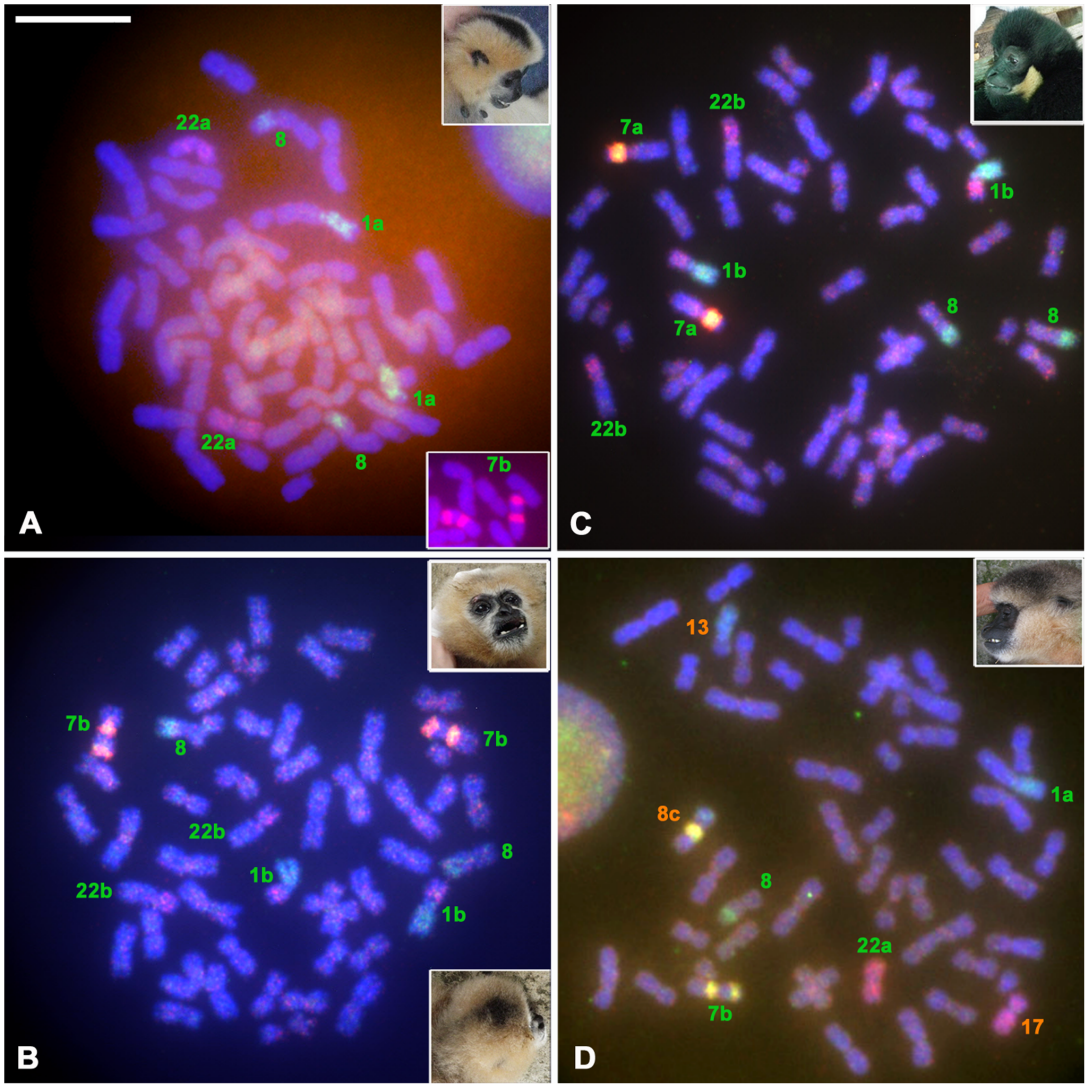
Chromosome paint analysis in light-cheeked gibbons. Chromosome paint analysis was performed with human chromosome paint probes 9 (green), 14 (red), and 22 (yellow) on the chromosomes of light-cheeked gibbons (*Nomascus*). **A)** *Nomascus leucogenys* (NLE, female northern white-cheeked gibbon) with karyotype 1a-7b-22a. **B)** *N. siki* (NSI, female southern white-cheeked gibbon) with karyotype 1b-7b-22b. **C)** *N. gabriellae* (NGA, male tuff-cheeked gibbon) with karyotype 1b-7a-22b. **D)** Female hybrid offspring consisting of NLE and *Hylobates lar* (HLA, lar gibbon) with karyotypes 1a-7b-22a and 8c, respectively. The photograph in the inset shows the morphology of each individual. Numbers and letters show the chromosome number and karyotype, in green for *Nomascus* spp. and in orange for *Hylobates lar*. Classification of species was performed using karyotypes described by Couturier and Lernould [28].

As shown above, AS location is a good marker to identify the chromosome sets of the two genera *Nomascus* and *Hylobates*, but it is not useful for identifying species within *Nomascus*. We tested the ability of painting probes for human chromosomes 9, 14, and 22 to provide information on classifying light-cheeked gibbons (not black faced: a subgroup of concolor gibbons) [36]. We analyzed the hybridization pattern of these paints on three captive individuals that were classified as *Nomascus* spp. by morphology in the Chiang Mai Zoo and Khao Kheow Open Zoo, Thailand. As shown above, although DAPI banding can also detect chromosome inversions, the three paint probes can more clearly distinguish between species of the light-cheeked gibbon group [28], [35]. The first individual showed karyotypes 1a-7b-22a, the specific marker for NLE (northern-white cheeked gibbon, Fig. 3A and inset). The second individual showed karyotypes 1b-7b-22b, the specific marker for NSI (southern white-cheeked gibbon, Fig. 3B and inset). Karyotypes 1b and 22b are produced by reciprocal translocation t(1;22). The third individual showed 1b-7a-22b, the specific marker for NGA (buff-cheeked gibbon, Fig. 3C and inset).

Finally, the hybrid offspring (*Nomascus*/*Hylobates*, Conlar) showed mixed karyotypes of 1a-7b-22a for NLE and 8c-13-17 for HLA (Fig. 3D). Incidentally, the previous hybrid offspring (*Hylobates*/*Nomascus*, Larcon) had 1a-7b-22a for NLE and 8b-13-17 for HLA [5]. The standard karyotype of NLE was described as 1a-7b-22a in the original paper [28]. The species name followed that used in previous papers [23], [37].

## Discussion

**Figure 4:**
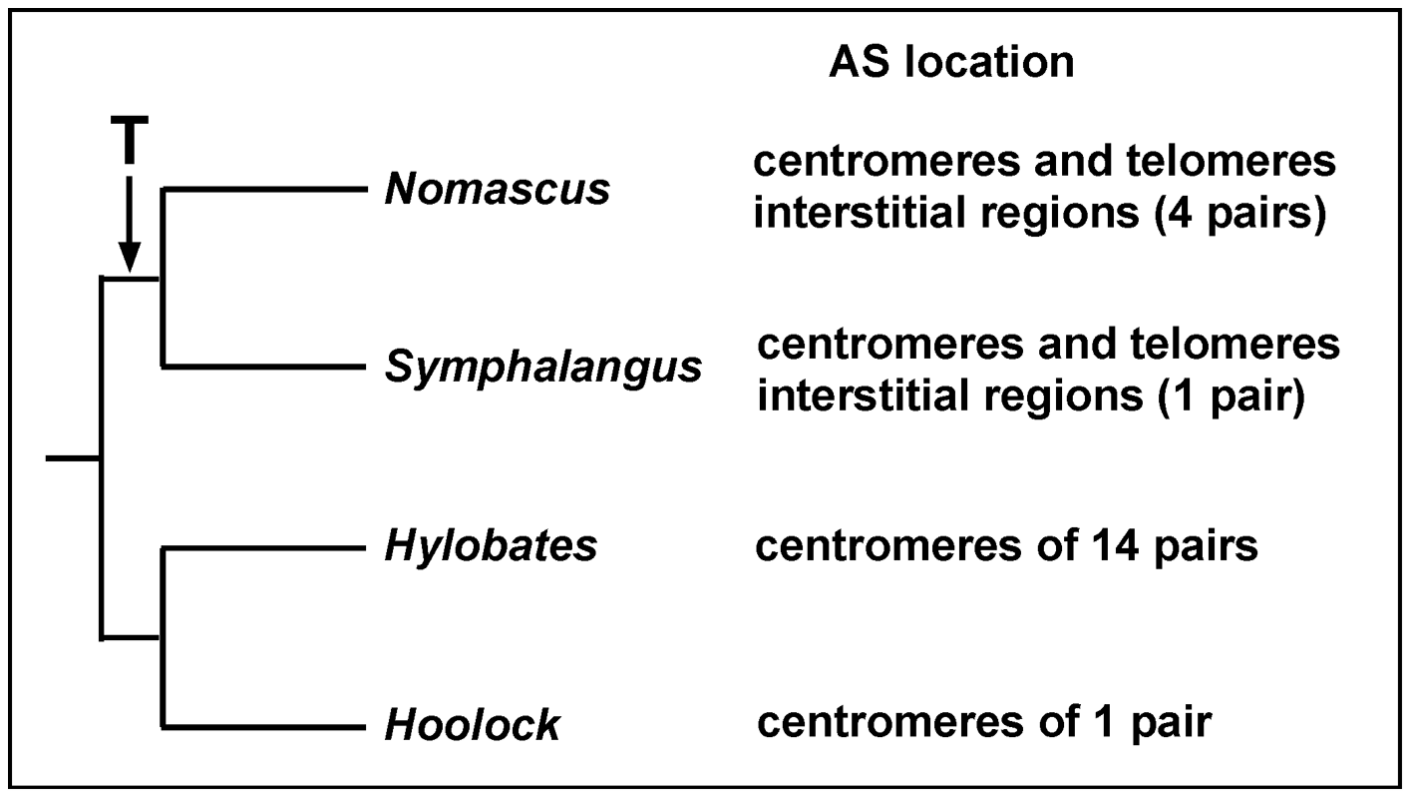
Qualitative phylogenetic differences in the small ape. The figure shows grouping of four genera of the small apes constructed by localization characteristics of AS. T, time period of transformation of the AS sequence to telomere regions. This topology was depicted with data from the present study and previous [32] studies.

The AS sequence was originally found at the centromeres of the African green monkey [38]; and is generally recognized as a major centromere DNA component [39]. More recently, however, we observed that small apes have AS sequences at both the centromeres and telomeres [31], [32], similar to those reported by Cellamare et al. [33]. Furthermore, the AS sequence allowed us to determine the phylogenic topology of the four genera of small apes [40]. The data divided them into two groups, the first group comprised a clade of *Nomascus* and *Symphalangus,* and the second group was composed of *Hylobates* and *Hoolock*. This grouping is in accordance with localization patterns of AS. The first group has AS blocks at both of the centromeres and telomeres, but the second group has AS blocks only at the centromeres [32]. The movement of AS to telomeres might have occurred in the ancestor of *Nomascus* and *Symphalangus* because other groups of hominoids do not have AS sequences at the telomere region [31]. Interstitial regions of AS blocks were specific in *Nomascus* and *Symphalangus*. These results allowed us to depict phylogenetic divergence at the level of chromosomal localization of AS (Fig. 4).

The pericentric inversion of chromosome 7 (7b) in the light-cheeked gibbons is a specific marker for classifying NLE. Carbone et al. [20] developed an excellent PCR technique using breakpoint primers spanned by a BAC clone (CH271-263C9) to detect inversion 7b. They found that the inversion is specific only for NLE because inversion 7b was observed as a PCR product only in NLE but not in NSI and NGA. However, our observations for chromosomes of the three species (NLE, NSI, and NGA) detected three karyotype morphs of 1a-7b-22a for NLE, 1b-7b-22b for NSI, and 1b-7a-22b for NGA, respectively, a finding that is consistent with previous results [28]. NLE7b and NSI7b might have a different break point with different sequences between each inversion, although further intensive research is needed. The PCR detection system for chromosome break points is quite useful as a cytotaxonomic technique for examining individuals who are represented only by DNA samples or extracted DNA [20].

In the case of small apes, cytotaxonomic information is a very helpful addition to morphological analysis because morphology is usually variable in all taxa. Here, we added a useful cytotaxonomic marker, AS, because as shown above the localizations are quite specific for each genus of small ape. Therefore, the localization can be used to detect the chromosome set of each genus. It is useful for identifying mixed genomes in intergeneric hybrid offspring and it is more accurate for detecting genus-specific chromosomes than C-band patterns (see Fig. 2 of [5]). In addition, the phylogenetic tree determined through AS sequences [40] is similar to that of the chromosome differentiation topology [12] and Y chromosome phylogeny [19]. Discovering more information on AS is also an opportunity to examine phylogenetic questions that are still controversial in small apes. In addition to AS, chromosomal rearrangements that were previously described were also useful as species diagnostic markers in the present study. The followings are diagnostic: inversions in chromosome 8 of *Hylobates* [11], translocation (WAT87/9) in *H. agilis* as a contrast to *H. albibarbis* [29], and inversion and translocation in *Nomascus* as mentioned above [20], [28], [35].

Arnold and Meyer [1] debated the source of incongruent phylogenetic relationships, in the evolution of gibbons and concluded that it was probably profoundly affected by natural hybridization. The intergeneric hybrid offspring observed in Japan and Thailand were named Larcon (HLA/NLE) and Conlar (NLE/HLA), respectively, from a combination of the species names of the mother and father. These individuals showed a different pelage pattern in the abdomen in both cases-Larcon is black, and Conlar is brown (data not shown). This difference in pelage pattern may also be caused by sexual dimorphism, which is substantial in the genus *Nomascus*. These situations may also be analogous to those in interspecific hybridizations that have occurred on the borders of natural populations [3]. Accordingly, hybridization appears to be a significant factor in the evolution and speciation of small apes. However, further comprehensive analyses combining different research fields will be required to thoroughly test this hypothesis and fully explain the complicated evolution of small apes.
